# Supplementary material for: Soil-Derived Bacillus pumilus Strains Demonstrate Antagonistic Activity Against Magnaporthe oryzae and Multiple Plant Growth-Promoting Traits
Source: Int J Mol Sci. 2026 Aug 12;27(16):7197. doi: 10.3390/ijms27167197 (PMC13513352; doi:10.3390/ijms27167197)
Supplement: Supplementary file 1 [file ijms-27-07197-s001.zip › Supplementary Table S1-S4, S6 and Figure S1.pdf]

Table S1. Genome-wide statistics.

Genome	Genome Length (bp)	GC Content (%)	Contigs	N50 (bp)	CheckM Completeness (%)
DC01	3,713,996	41.24	22	438,405	99.9
DC09	3,704,336	41.23	23	876,936	99.9
DC13	2,013,835	41.67	1	2,013,835	51.5

Table S2. Contig-level coverage statistics for DC01.

Contig ID	Average Fold Coverage	Contig Length (bp)	Percent Coverage	Median Fold Coverage	Standard Deviation
C1	295.8764	757258	100	295	45.07
C2	313.1126	519627	100	311	48.7
C3	290.2768	470408	100	288	47.76
C4	329.6988	438405	100	329	46.25
C5	337.3036	393932	100	337	44.7
C6	322.6397	370809	100	321	43.96
C7	312.3274	138985	100	311	44.4
C8	317.2909	151102	100	315	42.55
C9	321.6638	125451	100	317	71.88
C10	322.613	83767	100	321	46.38
C11	322.186	69547	99.9971	320	48.01
C12	338.2649	62349	100	332	110.16
C13	331.1697	55377	100	330	44.51
C14	324.1204	39271	100	324	45.47
C15	325.6708	18092	100	322	46.68
C16	352.0294	10994	100	352	41.09
C17	3052.894	3075	100	3100	362.15
C18	1769.1758	1809	100	1811	728.75
C19	451.1424	1215	100	371	192.42
C20	3029.4952	1565	100	3079	394.85
C21	258.0369	624	100	262	91.18
C22	503.1737	334	100	522	149.54

Table S3. Contig-level coverage statistics for DC09.

Contig ID	Average Fold Coverage	Contig Length (bp)	Percent Coverage	Median Fold Coverage	Standard Deviation
C1	194.8566	987792	100	193	36.12
C2	211.7628	876936	100	211	31.2
C3	219.0081	427049	100	218	32.99
C4	189.303	351221	100	187	34.48
C5	195.0522	285615	100	195	28.08
C6	208.7962	284228	100	206	42.9
C7	203.5785	245171	100	203	30.46
C8	222.8221	101078	100	223	32.37
C9	231.0078	62269	100	231	31.1
C10	229.5166	55492	100	228	32.96
C11	223.0009	18135	100	223	34.1
C12	2055.999	3048	100	2086	290.89
C13	303.5974	1915	100	250	134.9
C14	1893.1765	1139	100	1945	502.17
C15	170.5652	690	100	178	44.83
C16	387.0245	368	100	399	191.85
C17	1789.9432	458	100	2132	590.29
C18	164.0075	401	100	192	57.54
C19	270.7473	372	100	269	169.4
C20	287.4983	291	100	302	116.22
C21	155.498	249	100	170	60.68
C22	100.4836	213	100	94	44.66
C23	490.165	206	100	514	186.13

Table S4. Contig-level coverage statistics for DC13.

Contig ID	Average Fold Coverage	Contig Length (bp)	Percent Coverage	Median Fold Coverage	Standard Deviation
C1	15.5729	2013835	99.9881	15	14.11

Table S6. List of primers used for qRT-PCR.

Names	Primers Sequence (5′-3′)	Source
PAD4	Forward: GCACAAGTTTGAGCGCCATT<br>Reverse: ACTCCTTCACCCACAGGGAGTA	[40]
WRKY45	Forward: CGGGTAAAACGATCGAAAGA<br>Reverse: TTTCGAAAGCGGAAGAACAG	[40]
JAR1	Forward: TCTCCCCAGCCTTAACCGTA<br>Reverse: CTAAACGCGACGACAAACCC	[12]
EIL1	Forward: ACAATGCCACGATCATGGAG<br>Reverse: TCAGTAGTACCAATTCGAGC	[40]
PR1	Forward: CGTCTTCATCACCTGCAACTACTC<br>Reverse: CATGCATAAACACGTAGCATAGC	[40]
ERF1	Forward: CATATCACCTTGACGCCCCA<br>Reverse: ACCCTCACAAACTCACTCGG	[12]
EIN2	Forward: CAAGGAACCAGTGACAACCA<br>Reverse: GCAGTCGTCTCCGCAGTTAG	[43]
GAPDH	Forward: AAGCCAGCATCCTATGATCAGATT<br>Reverse: CGTAACCCAGAATACCCTTGAGTTT	[57]

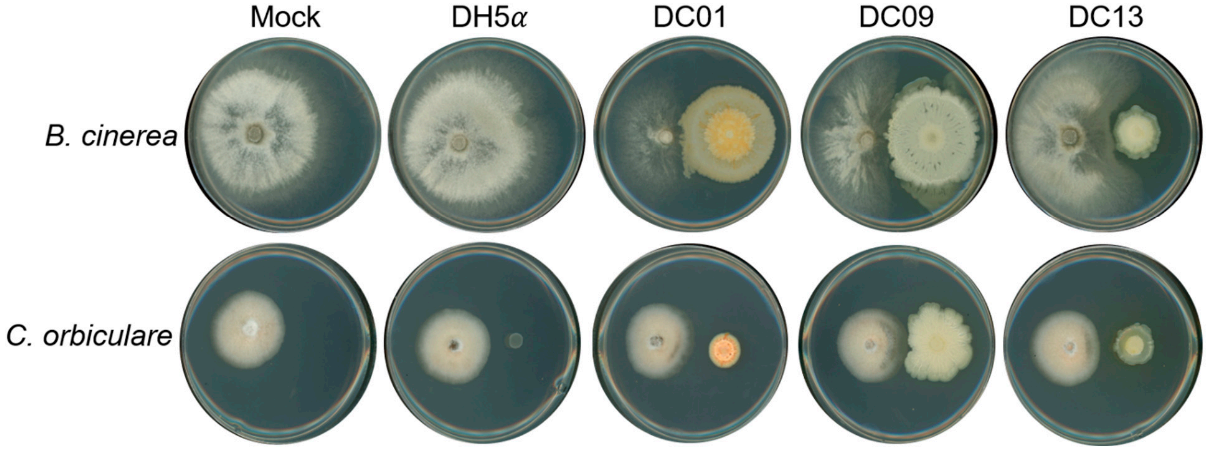

Figure S1. Antagonistic activity of *Bacillus* DC strains against *B. cinerea* and *C. orbiculare.* Dual culture assay plates showing fungal growth inhibition by DC strains compared to mock and DH5α controls. Plates were incubated at 25 °C, and images were taken at 5 dpi.
